# Mean Platelet Volume and Red Cell Distribution Width as a Diagnostic Marker in Acute Appendicitis

**DOI:** 10.5812/ircmj.10211

**Published:** 2014-05-05

**Authors:** Ceren Sen Tanrikulu, Yusuf Tanrikulu, Mehmet Zafer Sabuncuoglu, Mehmet Akif Karamercan, Nezih Akkapulu, Figen Coskun

**Affiliations:** 1 Department of Emergency Medicine, Ministry of Health, Erzurum Area Training and Research Hospital, Erzurum, Turkey; 2 Department of General Surgery, Ministry of Health, Erzurum Area Training and Research Hospital, Erzurum, Turkey; 3 Department of General Surgery, Faculty of Medicine, Suleyman Demirel University, Isparta, Turkey; 4 Department of Emergency Medicine, Faculty of Medicine, Gazi University, Ankara, Turkey; 5 Department of General Surgery, Ministry of Health, Mus State Hospital, Mus, Turkey; 6 Department of Chair of Emergency Medicine, Ankara Training and Research Hospital, Ministry of Health, Ankara, Turkey

**Keywords:** Appendicitis, Erythrocyte Indices, Blood Platelets

## Abstract

**Background:**

Acute appendicitis (AA) is one of the most common causes of emergent surgeries. Many methods are used for its diagnosis.

**Objectives:**

This study was conducted to investigate the diagnostic value of MPV and RDW in acute appendicitis.

**Patients and Methods:**

This study was a retrospective multi-center cross sectional planned study. The study included 260 patients operated for AA and 158 patients as the control group. Groups were compared in terms of MPV, RDW, white blood cell count (WBC), neutrophil predominance (NP) and platelet count (PC).

**Results:**

MPV was significantly lower in AA group, compared to the control group (P < 0.001). The best cut-off level for MVP in AA was ≤ 7.3 fL and the sensitivity, specificity, positive predictive value (PPV), negative predictive value (NPV) and overall accuracy ratio were 45%, 89.2%, 87.3%, 49.6% and 61.7%, respectively. There was no significant difference between the two groups in terms of RDW and platelet values.

**Conclusions:**

MPV is a routinely measured parameter in complete blood count (CBC) and requires no additional cost. It significantly decreased in AA, having a greater sensitivity and NPV when combined with WBC and NP.

## 1. Background

AA is one of the most common causes of emergent surgeries (1, 2). Currently, false diagnosis rates (15.3%) remain the same, equivalent to appendix rupture rates, despite all various laboratory and imaging techniques used (3). On the other hand, morbidity and mortality rates increase considerably in acute appendicitis, if surgical intervention is delayed (4). Taking a complete clinical history, physical examination and running laboratory tests, altogether do not raise the diagnostic power to 100% for early diagnosis of acute appendicitis (5, 6). Imaging modalities like ultrasonography and computerized tomography, as well as diagnostic laparoscopy and new laboratory tests have been increasingly used for fast and accurate diagnosis (7, 8). Therefore, easy, widely available, cheap, and time-saving new laboratory methods are needed.

Platelet count (PC) is a part of complete blood count (CBC) and one of the most commonly used laboratory tests. There are three CBC parameters related to platelets; plateletcrit (PCT), mean platelet volume (MPV) and platelet distribution width (PDW). MPV is the most well-known of these parameters and is a marker of platelet function and activation (9, 10). It has been studied as an inflammatory marker in various diseases and a decreased MPV has been reported in patients with ulcerative colitis, rheumatoid arthritis and ankylosing spondylitis (11, 12). There have been sparse studies done on decrease of MPV in AA (13, 14). RDW is an easy-to-measure part of CBC, showing variability of size of erythrocytes (15). Inflammation may impair red blood cell maturation through cell membrane damage, causing RDW to increase. In addition, several studies found strong correlations between RDW and inflammatory markers like C-reactive protein (CRP) and erythrocyte sedimentation rate (ESR) (16, 17).

## 2. Objectives

The aim of the present study was to investigate the diagnostic value of MPV and RDW in AA.

## 3. Patients and Methods

### 3.1. Study Groups and Study Design

This study was a multi-center basis retrospective and cross sectional planned study conducted after approving by the ethical committee of Turkey Ministry of Health, Ankara Research and Education Hospital Planning, and Coordination Committee at April 14, 2010 with the approval number of 2963. The study enrolled 260 AA and 158 control patients from Ankara Research and Education Hospital (600 beds, 12 sections, governmental, general, high direct admission and referral rate), Suleyman Demirel University Faculty of Medicine Hospital (400 beds, 14 sections, governmental, general, high direct admission), Erzincan Military Hospital (60 beds, four sections, military, normal direct admission and low referral rate) and Erzurum Regional Education and Research Hospital (700 beds, 18 sections, governmental, high direct admission and referral rate) between 1 January 2011 and 31 December 2011. Patients below 15 years of age, patients with previous abdominal surgery, pregnant women, patients with generalized peritonitis, patients with acute or chronic infections, unconscious patients with no cooperation, patients with accompanying diseases (like diabetes mellitus, hypertension, heart disease, vascular diseases, and cancer) and patients receiving medical therapy (analgesics, anticoagulant drugs, and oral contraceptives) were excluded from the study. Patients operated for acute appendicitis, 239 patients with histopathological diagnosis of acute appendicitis and 21 patients with histopathological diagnosis of normal appendix were included in the study. Pre-study power analysis could not be performed because the study was a multi-center retrospective one. The power of study was found to be 99% through post-study power analysis.

In patients operated with an initial diagnosis of AA, there were supporting findings in history, like right lower abdominal pain, appetite loss, nausea and vomiting and physical examination findings like guarding and rebound tenderness. Histopathologic examination was used as a basis for diagnosis of acute appendicitis; a normal appendix on histopathologic examination was a reason for exclusion. Control group was formed by patients with no symptoms, including patients admitted to outpatient check-up of centers. All patients underwent CBC analysis.

### 3.2. CBC Analysis

CBC analysis was performed on patients’ venous blood samples. Automated CBC devices were used, giving results with standard international normal values. WBC, NP, MPV, RDW and PC were evaluated. Normal values for all parameters were determined based on reference values accepted by hematology laboratories.

### 3.3. Histopathologic Assessment

Appendix samples obtained through surgery were grouped as appendicitis and normal, following histopathologic examination.

### 3.4. Statistical Analysis

Statistical analysis was performed using SPSS 15.0 software (SPSS Inc., Chicago, IL, USA). Distribution of data was determined by Kolmogorov-Smirnov test. Continuous variables were expressed as mean ± SD and categorical variables as frequency and percent. Continuous variables were compared using independent sample t-test or Mann-Whitney U-test and categorical variables were compared using Pearson’s Chi-square test for two groups. Receiver operating characteristic (ROC) curve analysis was used to detect optimal cut-off levels for MPV and RDW. Specificity, sensitivity, positive predictive value, negative predictive value and overall accuracy rates were calculated for MPV and RDW. In addition Youden’s index was calculated for optimized of overall rates accuracy. A P value < 0.05 was considered statistically significant.

## 4. Results

The demographic characteristics of study and control populations are shown in Table 1. According to these results, 260 patients were enrolled in the AA and 158 patients in the control group. Both groups were similar in terms of age, AA group having a mean age of 31.8 and control group having a mean age of 32.2. Among patients with AA, 109 (42%) were women and 151 (58%) were men and in the control group 86 (54%) of patients were women and 72 (46%) were men. There were differences between patients based on the population density and the hospital capacities of the study centers. The histopathology results are summarized In Table 2. Based on these results, 239 (92%) out of 260 patients with AA were also histopathologically diagnosed with acute appendicitis, while 21 (8%) patients had normal appendix. According to these results, negative appendectomy rate was 8%.

**Table 1.** Demographic Characteristics of Groups ^a^, ^b^

	Acute Appendicitis (n = 260)	Control Group (n = 158)	P value
**Age, y**	31.8 ± 12.4	32.2 ± 10.5	0.190
**Gender**			0.013
Female	109 (42)	86 (54)	
Male	151 (58)	72 (46)	
**Centers**			< 0.001
AEAH	175 (67)	72 (46)	
SDU	39 (15)	18 (11)	
EAH	26 (10)	28 (18)	
EBEAH	20 (8)	40 (25)	

^a^ Abbreviations: Abbreviations: AEAH, Ankara education and research hospital; EAH, Erzincan military hospital; EBEAH, Erzurum area education and research hospital; SDU, Suleyman Demirel university.

^b^ Data are presented in Mean ± SD or No. (%).

**Table 2.** Histopathology Assessment Results ^a^

Histopathological Result	Result
**Acute appendicitis**	239 (92)
**Parasitic appendicitis**	3
**Chronic appendicitis**	1
**Carcinoid tumor**	1
**Normal appendix**	21 (8)

^a^ Data are presented in No. (%).

**Figure 1.**
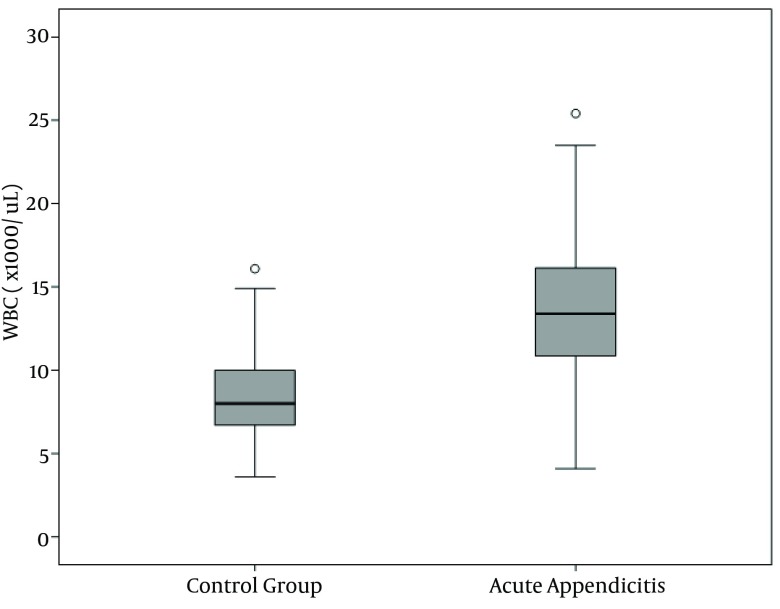
White Blood Cell Count Distribution in the Groups

**Figure 2.**
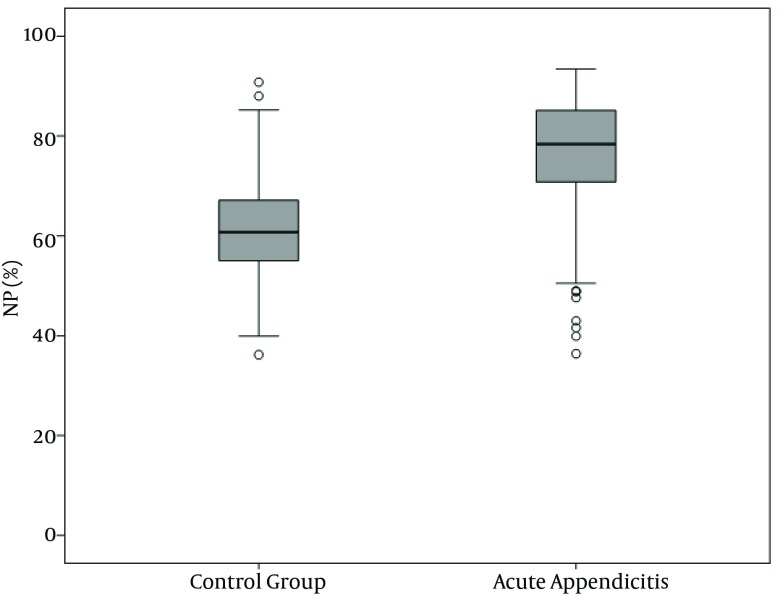
Neutrophil Percent Distribution in the Groups

**Figure 3.**
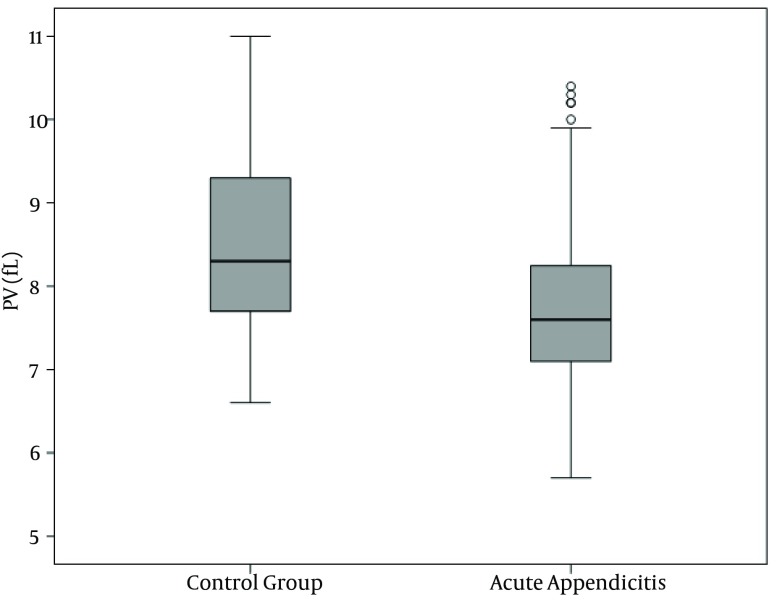
Mean Platelet Volume Distribution in the Groups

**Figure 4.**
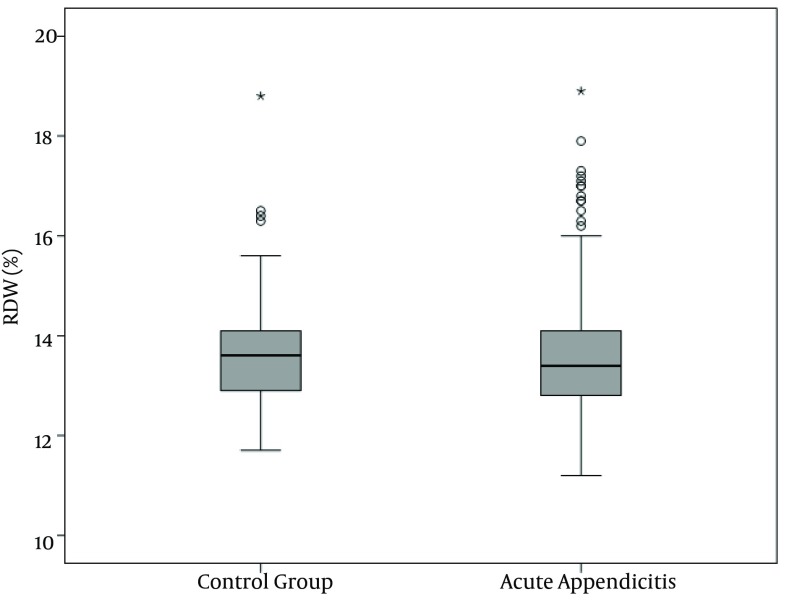
Red Cell Distribution Width Distribution in the Groups

**Figure 5.**
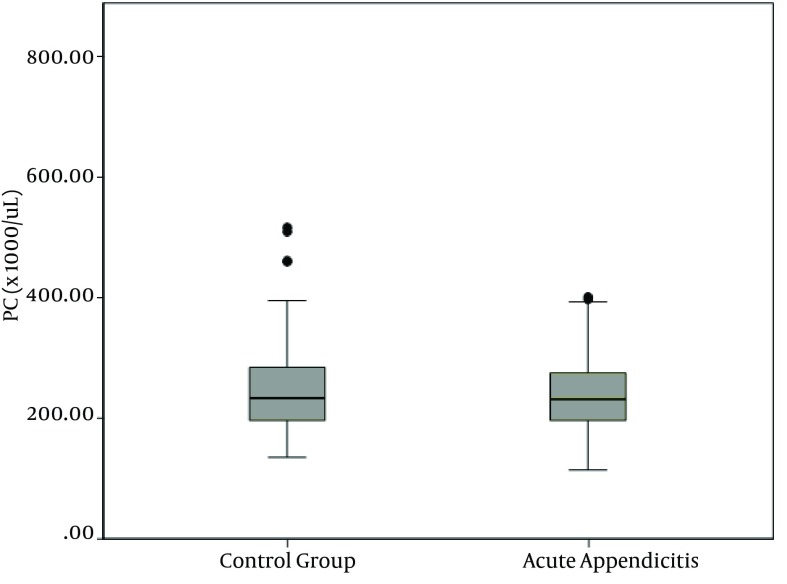
Platelet Count Distribution in the Groups

WBC, NP, MPV, RDW and PC results are demonstrated in Figures 1, 2, 3, 4 and 5 and Table 3. There was a significant difference between groups in terms of WBC (P < 0.001); 199 (77%) out of 260 patients in AA group having a high WBC count vs. 12 (8%) out of 158, in the control group. Both groups differed significantly in terms of NP results (P < 0.001); 199 (77%) out of 260 patients in the AA group having positive NP, vs. 15 (%9) out of 158 patients in the control group. MPV results differed significantly between the two groups (P < 0.001); 114 (44%) out of 260 patients in the AA group having low MPV levels vs. 15 (9%) out of 158 patients in the control group. RDW and PC levels were similar in both groups without statistically significant difference (P = 0.478 for RDW, P = 0.925 for PC). The sensitivity and specificity pertaining to CBC parameters, as well as the ROC curve of MPV and RDW are demonstrated in Table 4 and Figure 2. Sensitivity, specificity, PPV, NPV and overall accuracy rates for WBC (> 11 × 10^9^/L) and NP (> 70%) were 76.2%, 90.5%, 93%, 69.8% and 81.6%, and 75.3%, 90.5%, 92.6%, 69.1% and 81.1%, respectively. Sensitivity, specificity, PPV, NPV and overall accuracy rates for MPV levels were 45%, 89.2%, 87.3%, 49.6% and 61.7% and for RDW levels were 18.5%, 92.4%, 80%, 40.8% and 46.4%, respectively. The Youden’s index for WBC, NP, MPV and RDW were 0.666, 0.658, 0.342 and 0.109, respectively. In ROC analysis results, cut-off values, the best sensitivity and specificity for WBC, NP, MPV and RDW were 10.6 × 10^9^/L (84%-91%), 70.2% (81%-90%), ≤ 7.3 fL (78%-67%) and >14.5% (47%-57%), respectively. The area under curve (AUC) values for WBC, NP, MPV and RDW were 87.6%, 86.5%, 71.5%, and 52.1%, respectively.

**Figure 6.**
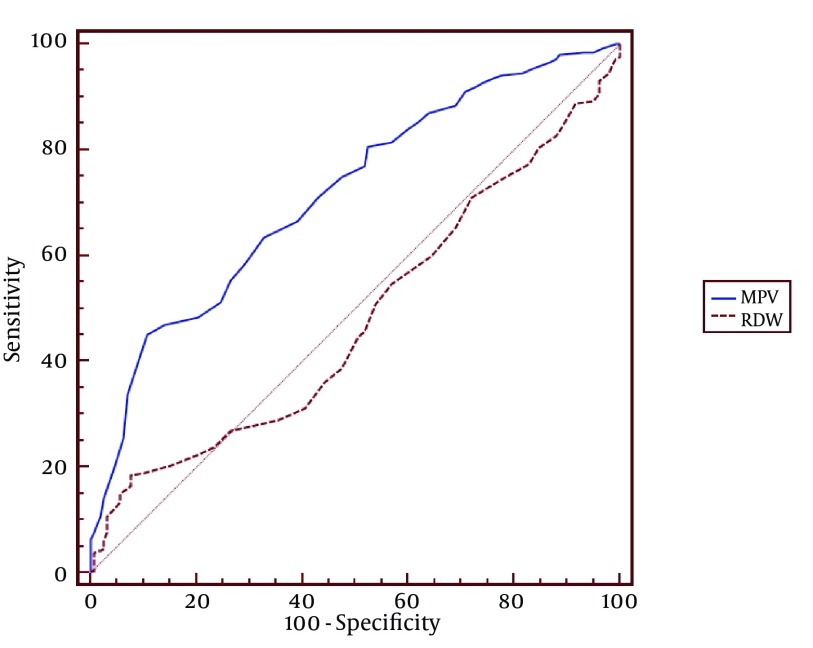
Receiver Operating Characteristic Curves of Mean platelet volume and Red Cell Distribution Width

**Table 3.** Comparison of Complete Blood Count Parameters Across Groups ^a^

	Acute Appendicitis (n = 260)	Control Group (n = 158)	P value
**WBC, × 10** ^**9**^ **/L**	13.48 ± 3.92	8.28 ± 2.17	< 0.001
**NP, %**	76.60 ± 11.49	60.88 ± 9.65	< 0.001
**MPV, fL**	7.75 ± 1.24	8.49 ± 0.97	< 0.001
**RDW, %**	13.59 ± 1.24	13.57 ± 0.96	0.478
**PC, × 10** ^**9**^ **/L**	251.24 ± 74.64	251.53 ± 70.77	0.925

^a^ Abbreviations: MPV, mean platelet volume; NP, neutrophil predominance; PC, platelet count; RDW, red blood cell distribution width; WBC, white blood cell.

**Table 4.** Overall Accuracy Rates of Complete Blood Count Parameters (%) ^a^

	Sensitivity	Specificity	PPD	NPD	Accuracy	Youden’s Index
**WBC**	76.2	90.5	93.0	69.8	81.6	0.666
**NP**	75.3	90.5	92.9	69.1	81.1	0.658
**MPV**	45.0	89.2	87.3	49.6	61.7	0.342
**RDW**	18.5	92.4	80.0	40.8	46.4	0.109

^a^ Abbreviations: NPV, negative predictive value; PPD, positive predictive value; RDW, red blood cell distribution width; WBC, white blood cell.

## 5. Discussion

This study investigated diagnostic values of MPV and RDW in AA. There is a limited number of studies in literature showing MPV changes in AA, whereas there is no study on the RDW (13, 14). MPV is a marker of platelet function and activation, can be easily measured in routine CBC and has been increasingly used, particularly in diagnosis of inflammatory diseases (7, 8). Since studies demonstrated higher MPV values in chronic obstructive pulmonary disease, diabetes mellitus and myocardial infarction, patients with such systemic diseases were excluded (18, 19).

Kisacik et al. (13) demonstrated that MPV levels decreased in rheumatic diseases like rheumatoid arthritis and ankylosing spondylitis. Yuksel et al. (12) reported a decreased MPV level in ulcerative colitis. Similarly, Danese et al. (20) found a decreased MPV level in inflammatory bowel disease and suggested that the decrease stems from sequestration or consumption of large activated platelets due to intestinal vascularization. Makay et al. (21) found no significantly different MPV levels in control and patient groups but a decreased MPV level was detected at the time of attack, in a patient group in a pediatric patient cohort of familial Mediterranean fever. Albayrak et al. (14) detected a significantly lower MPV level in patients with AA, compared to the control group. Another study by Bilici et al. (15) found similar results in children with AA.

Findings of the present study showed similar results to that of lbayrak et al. (14) and Bilici et al. (15); MPV level was significantly lower in the AA group, compared to controls (P < 0.001). There was no significant difference between groups in terms of platelet numbers (P = 0.925), consistent with the literature. In various studies about diagnosis of AA, the sensitivity of WBC was 85.8%, 97.8% and 76.5%, specificity was 31.9%, 55.6% and 90.8%, the sensitivity of neutrophil count was 87.2%, 98.9% and 68.6%, specificity was 33.1%, 38.9% and 86.4% (13, 21, 22). We found sensitivity, specificity, PPV, NPV and overall accuracy rates for WBC; 76.2%, 90.5 %, 93.0%, 69.8% and 81.6%, respectively, consistent with the literature. In addition, sensitivity, specificity, PPV, NPV and overall accuracy rates of NP were 75.3%, 90.5%, 92.9%, 69.1% and 81.1%, respectively. ROC analysis revealed cut-off levels of WBC, NP, MPV and RDW and the best sensitivity and specificity of these values to be 10.6 × 10^9^/L (84%-91%), 70.2% (81%-90%), ≤ 7.3 fL (78%-67%) and > 14.5% (47%-52%), respectively. Our study detected a higher sensitivity and a lower specificity of MPV, compared to the findings of Albayrak and colleagues study (14). In addition, AUC was found to be 0.72, which was lower than 0.86, found by Albayrak and colleagues. RDW is a parameter of the distribution extent of erythrocytes in circulation. It is routinely studied in CBC. Studies on different populations recently suggested an increased mortality with increasing RDW levels. Apart from etiologies of anemia, oxidative stress and inflammation are known to influence RDW levels by altering maturation of erythrocytes via affecting cell membrane (16, 23). In addition, RDW is known to exhibit a strong correlation with markers like CRP and ESR in inflammatory conditions (17). Cakal et al. (24) detected a significant increase in RDW, particularly at the attack periods of inflammatory bowel disease. We did not find a significant difference between control group and AA group.

To conclude, WBC elevation and presence of NP support the diagnosis of AA, when suspected in patients through history and physical examination. MPV analysis is studied in routine CBC, has no additional cost and may be used as a supportive test in addition to other parameters. We believe that further studies with larger numbers of patients are essential to establish RDW as a diagnostic or ancillary test.
